# Ustekinumab Drug Clearance Is Better Associated with Disease Control than Serum Trough Concentrations in a Prospective Cohort of Inflammatory Bowel Disease †

**DOI:** 10.3390/pharmaceutics17020187

**Published:** 2025-02-02

**Authors:** Andres J. Yarur, Thierry Dervieux, Ryan Ungaro, Elizabeth A. Spencer, Alexandra Bruss, Lizbeth Nunez, Brandon Berens, Séverine Vermeire, Zhigang Wang, John C. Panetta, Erwin Dreesen, Marla C. Dubinsky

**Affiliations:** 1 Cedars Sinai Medical Center, Los Angeles, CA 90001, USA; andres.yarur@cshs.org; 2 Prometheus Laboratories, San Diego, CA 92121, USA; 3 Mount Sinai Medical Center, New York, NY 10029, USA; ryan.ungaro@mssm.edu (R.U.); elizabeth.spencer@mssm.edu (E.A.S.); 4 Medical College of Wisconsin, Milwaukee, WI 53226, USA; abruss@mcw.edu (A.B.); lnunez@mcw.edu (L.N.); bberens@mcw.edu (B.B.); 5 KU Leuven, Department of Gastroenterology, Pharmacometrics, 3000 Leuven, Belgium; severine.vermeire@uzleuven.be (S.V.); zhigang.wang@kuleuven.be (Z.W.); erwin.dreesen@kuleuven.be (E.D.); 6 St Jude Children’s Research Hospital, Memphis, TN 38016, USA; carl.panetta@stjude.org

**Keywords:** ustekinumab, inflammatory bowel disease, pharmacokinetics, clearance, therapeutic drug monitoring

## Abstract

**Background/Objectives**: This study aimed to compare the association of ustekinumab (UST) drug clearance (CL) and trough drug concentrations with disease activity in patients with inflammatory bowel diseases (IBDs). **Methods**: A prospective cohort of 83 patients with IBD receiving maintenance therapy with 90 mg subcutaneous UST was analyzed using Bayesian PK modeling. UST concentrations and antibodies to UST (ATU) were collected at the trough and measured using a drug-tolerant homogenous mobility shift assay (HMSA). CL was estimated using Bayesian estimation methods with priors from a previous population pharmacokinetic study specifically reparametrized using HMSA. Outcomes were combined clinical and biochemical remission and endoscopic healing index (EHI) score, a validated marker of endoscopic active disease in IBD. Statistical analysis consisted of linear and nonlinear mixed effect models for repeated time-to-event analysis. **Results**: A total of 83 patients with IBD were enrolled (median age 42 years, 52% female) and evaluated across 312 dose cycles (median follow-up: 279 days, median of 3 cycles/patient). Median concentrations and CL were 5.0 µg/mL and 0.157 L/day, respectively. Most patients (89%) were exposed to other biologics before starting UST, which was associated with lower rates of clinical and biochemical remission (*p* = 0.01). Longitudinal changes in concentrations were not associated with remission (*p* = 0.53). Conversely, higher CL was associated with a lower likelihood of remission (*p* < 0.01). EHI > 50 points (endoscopic active disease, *n* = 303 cycles) was associated with higher UST CL (*p* < 0.01). **Conclusions**: UST CL was more strongly associated with clinical and biochemical outcomes than trough concentrations, highlighting its potential role in therapy optimization.

## 1. Introduction

Ustekinumab (UST) is a monoclonal antibody targeting the IL-12/23 inflammatory pathway and is effective for the treatment of moderate to severe Crohn’s disease [CD] and ulcerative colitis [UC]) [1,2]. These two conditions fall under an umbrella known as inflammatory bowel diseases (IBDs) [3]. IBD is characterized by inflammation of the gut with symptoms consisting of abdominal pain, diarrhea, rectal bleeding, fatigue, and weight loss, all contributing to poor quality of life and disability [1,2].

UST has positioned itself as an important drug within the IBD therapeutic armamentarium for both induction and maintenance of remission. However, as seen with anti-tumor necrosis factor-α (TNF-α) monoclonal antibodies [4], a significant proportion of patients do not respond to UST or lose response after experiencing a benefit. Identifying mechanisms of non-response, including pharmacokinetics (PKs), is important as dose optimization could recapture some of these treatment failures [5].

In clinical practice, serum trough UST concentrations have been associated with efficacy and are a metric of the exposure-effect relationship in both IBD [6,7,8] and psoriasis [9,10]. Some data support the potential value of conducting therapeutic drug monitoring through the use of UST concentrations (TDM) [6,11,12]. In their meta-analysis, Vasudevan et al. [6] identified 14 observational studies in IBD where median UST serum concentrations were found to be higher in patients in clinical remission as compared to those with active disease. However, serum UST trough concentrations have limited predictive value, prompting interest in alternative PK metrics such as drug clearance. This is partly driven by the heterogeneity of the disease [13] and also due to the difference seen in drug assays commonly used to measure drug concentrations. This makes the interpretation of results across assays and studies difficult. For example, concentrations using the fluid phase homogenous mobility shift assays (HMSAs) are higher when compared to solid phase assays, and thus, cutoff values are not interchangeable between techniques [14]. We previously established the performances of UST concentrations by HMSA in patients with IBD and demonstrated that UST trough serum concentration above 4.5 μg/mL after 26 weeks of therapy was associated with biochemical and endoscopic response [15].

It follows that serum UST trough concentrations can help guide clinical management, and more recent reports have established that exposure monitoring might be beneficial in achieving improved outcomes [16,17]. Particularly, measuring exposure can identify those patients with undetectable or very low UST concentrations (below <1 µg/mL). Unfortunately, in most other scenarios, the measured trough concentration does not appear to be very helpful in predicting clinical outcomes [18], and there is a need to identify and validate additional PK metrics that could potentially be used to guide clinical practice.

More recently, drug clearance (CL), the volume of serum completely cleared from the drug as a function of time, is better associated with response to infliximab and adalimumab [19,20,21] compared to concentrations alone. Drug CL reflects disease (i.e., the inflammatory burden [22]) and individual characteristics (i.e., the formation of anti-drug antibodies [23,24]) and might therefore provide more clinically relevant information when compared to concentrations alone. In the first study of 145 pediatric patients with IBD [20], the impact of CL on IFX outcome was demonstrated, and a lower likelihood of clinical and biochemical remission during both induction and maintenance was observed with higher CL. During induction, higher CL (>0.294 L/day) measured before the third (hazard ratio = 1.5 95%CI: 1.0 to 2.3) and fourth infusion (HR = 2.1 95%CI: 1.3 3.2) predicted a lower probability of achieving clinical and biochemical remission during maintenance. UST CL during maintenance was also better associated with disease control when compared to UST than concentrations during the maintenance period. Similarly, in a second study of 237 patients who were receiving ADA [21], median CL was higher in patients with persistent active endoscopic disease compared to those who had achieved endoscopic remission (0.326 L/day vs. 0.247 L/day, respectively) with no significant difference seen in ADA concentration (median 11.7 µg/mL vs. 9.3 µg/mL, respectively). These suggest that drug CL may be a superior predictor of therapeutic response to anti-TNF-α agents than concentrations. This concept of CL associating with therapeutic outcomes might also apply to other monoclonal antibodies. As other biologics with alternative mechanisms of action have been introduced to treat IBD, there is a need to understand how we can better predict response and identify mechanisms of PK nonresponse that lead to interventions to optimize outcomes. There is a paucity of data associating UST CL during induction and maintenance with therapeutic outcomes in IBD. One study [25] reported higher UST CL measured during induction at week 2 (0.25 L/day vs. 0.20 L/day) and week 6 (0.27 L/day vs. 0.21 L/day) with worse endoscopic response at week 24.

The aim of this study was to compare the association between either UST CL or serum trough concentrations and clinical outcomes in a real-world population receiving the drug for the treatment of IBD. We demonstrate that higher CL is associated with worse therapeutic outcomes in contrast to UST trough concentrations poorly associated with disease control.

## 2. Materials and Methods

### 2.1. Patients and Settings in Prospective Study

This was a prospective cohort study performed in two centers from the United States: Medical College of Wisconsin (Milwaukee, WI, USA) and Mount Sinai Hospital Icahn School of Medicine (New York, NY, USA). The study included patients with a confirmed diagnosis of CD or UC on UST maintenance therapy with subcutaneous UST 90 mg every 4 to 8 weeks after receiving one IV loading dose as per label. All patients (or their legal guardians) signed informed consent, and the study was approved by each institutional review board. Patients with an ostomy or a J pouch were excluded.

### 2.2. Sample Collection and Laboratory Measurements

Up to 6 trough blood specimens (immediately before the subcutaneous injection) and clinical assessments were collected sequentially throughout the follow-up. All patients must have been on UST for at least 16 weeks and have received the first weight-based IV loading infusion followed by two consecutive doses at 8-week intervals. Venous blood was collected in serum separator tubes, allowed to clot at room temperature and spun. Isolated serum was shipped within 48 h of collection to the clinical laboratory at Prometheus Labs (San Diego, CA, USA) and stored at −80 °C until testing. Pre-analytical studies have shown that UST and antibodies to UST (ATU) in serum are stable at room temperature for 7 days (data on file, Prometheus Laboratories).

Trough serum, UST concentrations, and ATU were measured using a validated drug tolerant HMSA (size exclusion liquid chromatography coupled with fluorometric detection) through the follow-up in the CLIA/CAP New York State accredited clinical PK laboratory at Prometheus Laboratories (San Diego, CA, USA) [15,26,27]. Upon receipt, UST (lower limit of quantification [LLOQ] = 0.9 µg/mL; upper limit of quantification [ULOQ] = 25 µg/mL) and ATU concentrations (>1.6 U/mL indicating positive ATU status) were measured as per standard operating procedures currently in use.

Additionally, albumin (ALB) was measured using immunoassay (IMMAGE 800, Beckman Coulter, Brea, CA, USA). Samples were also analyzed for the endoscopic healing index (EHI) score, a multianalyte assay algorithm consisting of 13 biomarkers from serum (including CRP and 12 other proteins) and validated to be a noninvasive surrogate marker of endoscopic inflammation in CD [28] and UC [29]. The EHI score ranges from 0 to 100; a score > 50 points is 30% sensitive and 87% specific for the presence of active endoscopic disease in CD (simple endoscopic score CD > 6 points) [28]. In UC, an EHI > 50 points is 77% sensitive and 88% specific for endoscopic active disease (Mayo Endoscopic Subscore > 1 point) [29]. All clinicians, coordinators, and testing personnel were blinded to the test results throughout the study period.

### 2.3. Bayesian Priors and Population PK Model Reparameterization Using HMSA Format

The individual UST CL estimates were determined using Bayesian estimation methods. The Bayesian priors were based on data from the previously published population PK study [30] (training cohort) which used samples evaluated with an enzyme-linked immunoassay (ELISA). To be consistent with the HMSA used in the current study, the samples from the previous study were re-assayed using the HMSA. Specifically, the sera from consented CD individuals were anonymized, stored at −80 °C, and shipped (n = 308 observations from 65 patients).

Next, the population PK model was re-parameterized using the re-assayed UST and ALB concentrations. Specifically, these data were fit using nonlinear mixed effect modeling (via NONMEM version 7.5) using the same model structure initially reported [30]. UST concentrations below the LLOQ (0.9 µg/mL) or above the ULOQ (25 µg/mL) were treated as censored using the M3 method. The population parameters estimated included CL (L/day), the volume of distribution in the central and peripheral compartments (V1 and V2, respectively, expressed in L), and intercompartmental CL (Q, L/day). Absorption (ka) was fixed to 0.2 (L/days). A combined additive and residual error model was used.

Since there were no ATU-positive individuals in this cohort, the effect of ATU status on CL was determined using the Prometheus PK database of 11,372 patient specimens (average UST concentration 6.7 µg/mL), of which 57 specimens were ATU positive. Specifically, all the population PK parameters estimated with the above data (from [30]), were fixed, and only the effects of ATU on UST Clearance were estimated.

The UST CL was estimated using Bayesian parameter estimation. Specifically, the fixed and random effects of the PK parameters, along with the covariate effects of ALB and ATU from the re-parameterized models of the previous studies described above, were all fixed, and the empirical Bayesian estimates (i.e., post hoc individual pharmacokinetic parameter estimates) were estimated for the current study using Monolix (R2023a). This was accomplished by estimating the conditional distributions of the model parameter (Cl), generated for each patient using Markov Chain Monte Carlo simulations (Metropolis–Hastings algorithm), and sampling (n = 100) from that distribution.

The analytical validation of the population PK model and estimated CL was established using different serum specimens from different patients enrolled in the US perspective cohort (90 mg every 8 weeks and under therapy for at least 16 weeks). Intra-day variability for CL was established using 8 specimens, with each specimen processed the same day 8 consecutive times, and the inter-day variability was established using the same specimens processed on 5 consecutive days.

### 2.4. Outcome Measures

The primary outcome was a combination of clinical and biochemical remission defined as a Harvey Bradshaw Index < 5 points (in CD), a partial Mayo score < 2 points (in UC), and a normal CRP (<3 mg/L) [20]. The Harvey Bradshaw Index is a CD disease activity index comprising 5 domains, including patients’ general wellbeing, abdominal pain, number of liquid stools per day, abdominal mass, and presence of extra-intestinal complications, all scored individually to produce the score [31]. The partial Mayo score is a disease activity index used in UC, and that includes the total Mayo Score, which uses three noninvasive components: stool frequency, rectal bleeding, and Physician’s global assessment [32]. The secondary outcome was an EHI > 50 points, indicating a higher likelihood of endoscopic active disease as described above.

### 2.5. Statistical Analysis

The hypothesis was that higher UST CL and lower concentrations would associated with worse disease control during maintenance therapy of IBD. Group comparisons were performed using Fisher’s Exact test. Changes in clinical and biochemical remission status (categorial variable) or EHI score (categorical variable corresponding to EHI > 50 points) as a function of PK metrics (UST CL and concentrations) and prior use of biologics were analyzed using logistic regression models via Monolix (Lixoft, 2023R1, Paris, France). These models estimated the probability of the categorical outcome as a function of the covariates (e.g., UST CL). Since there were multiple PK studies per patient, nonlinear mixed effects modeling, which accounts for both the variability within and between individuals, was used. *p* values below 0.05 were considered statistically significant.

## 3. Results

### 3.1. PK Model Reparameterization Using HMSA and Bayesian Priors

As expected, reported UST concentrations in the training cohort [30] measured using HMSA (median: 5.5 µg/mL IQR: 3.1–11.4 µg/mL) were 1.5-fold higher than those recovered using the solid phase ELISA assay (median: 3.6 g/mL IQR: 1.7–7.4 µg/mL) (slope = 1.5 [95%CI: 1.4–1.1], intercept = 0.5) with good correlation (R^2^ = 0.97). Table 1 presents the parameter estimates for the re-parameterized model of the training cohort using the HMSA UST and ALB concentrations, the quantification of the ATU effects using the data from the Prometheus PK database, and the final Bayesian priors to be used in this study.

The population estimate of CL was lower (0.161 L vs. 0.215 L/day), and bioavailability was higher (0.735 vs. 0.624) with the HMSA, as compared to the ELISA assay, a finding consistent with the higher concentrations recovered using HMSA versus the solid phase assay ELISA assay. Additionally, based on the second cohort of individuals that included positive ATU studies from the Prometheus clinical database (n = 11,372), UST clearance was 1.76X higher in positive ATU specimens. All estimates from the fully re-parameterized PK Model, including the covariate effects of ALB and ATU, were then fixed and used as Bayesian priors in the current study (Table 1).

The intra-day CV for UST concentration and CL estimated using the population PK model were below 10% (acceptable performances are below 15% CV in the clinical PK laboratory) for each of the specimens tested (Table 2), and similar performances were observed with interday CVs (8 specimens processed on 5 separate days) (Table 3).

### 3.2. Patient Characteristics

The study enrolled 83 patients (52% female, 78 CD, median age 42 years [IQR: 28–57] evaluated across 312 UST dose cycles (median of 3 cycles per patient [range 1–6], n = 77 cycles with every 4 weeks dosing schedule). Patient characteristics are presented in Table 4. Most patients were exposed to a prior biologic therapy (89%, 74/83).

In a total of 312 maintenance cycles, the median UST trough concentration and estimated CL were 5.0 µg/mL and 0.157 L/day, respectively. None of the samples were ATU-positive. Most patients (76/83) had poor disease control during UST maintenance, and 55% (46/83) never achieved clinical and biochemical remission at any of the time points. Only 8% of patients (7/83) had sustained clinical and biochemical (on all cycles). The median EHI measured in 303 cycles was 33 points (IQR: 23–43), with 18% having EHI above 50 points.

### 3.3. Impact of Prior Biologics on Outcome and Pharmacokinetic Metrics

**Figure 1:**
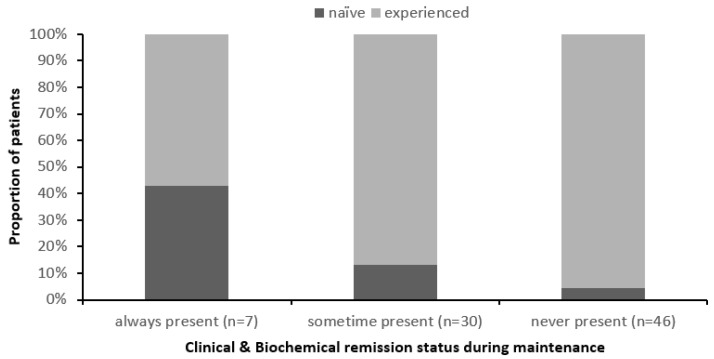
**Prior biological usage and achievement of clinical and biochemical remission during maintenance of IBD** (*p* = 0.008). Naïve corresponds to patients who received ustekinumab as the first biologic; experienced corresponds to patients who had received other biologic therapy before commencing ustekinumab.

Prior biologic usage was associated with active disease. Specifically, a higher proportion of biologic-experienced patients were in the group who never achieved remission (96%, 44/46) than in the group of patients who were sometimes (87%, 26/30) or always in remission (57%, 4/7) (*p* = 0.008) (Figure 1).

Consistent with these observations, prior biologic usage was significantly associated with a lower probability of remission (*p* = 0.004), with time of treatment having little impact on the probability of remission (*p* = 0.158), where the probability of having clinical and biochemical remission at 279 days (median follow up) was 66% in the absence of prior biologics compared to 14% with prior biologics (Table S1).

### 3.4. Impact of PK Metric on Outcomes

**Figure 2:**
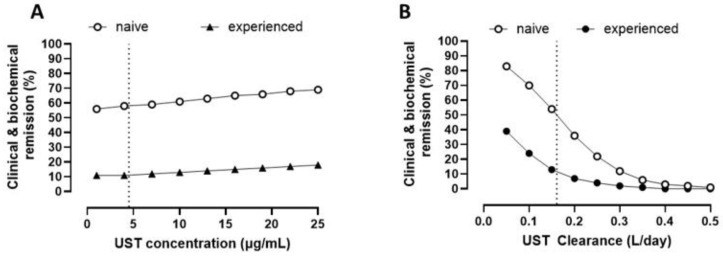
**Probability of clinical and biochemical remission by ustekinumab concentration and clearance**. (**A**) Clinical and biochemical remission and UST concentrations: prior biologics, *p* = 0.005; concentrations, *p* = 0.533; dotted line corresponds to 4.5 µg/mL; (**B**) clinical and biochemical remission and UST CL: prior biologics *p* = 0.013; CL *p* < 0.001. The dotted line corresponds to 0.16 L/day the typical value of the population PK model re-parameterized using HMSA. Estimates are provided in Table S1.

UST concentrations were not associated with achieving clinical and biochemical remission (Figure 2A; *p* = 0.533). There was little change in the probability of remission across the range of concentrations observed with this analysis. In contrast, both higher CL and exposure to prior biologics were significantly associated with a lower probability of clinical and biochemical remission (*p* < 0.001 and *p* = 0.013, respectively; Figure 2B).

**Figure 3:**
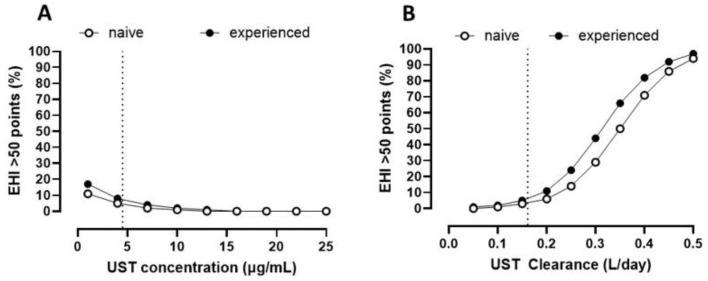
**Association of Endoscopic Healing Index (EHI) scores above 50 points and pharmacokinetic metrics**. (**A**) EHI > 50 and ustekinumab concentrations: prior biologics, *p* = 0.731; concentrations, *p* = 0.001; (**B**) EHI greater than 50 and ustekinumab CL: prior biologics, *p* = 0.659); CL; *p* < 0.001. Dotted lines corresponds to UST concentration cutoff at 4.5 µg/mL (**A**) and 0.161 L/day (**B**). Estimates are provided in Table S1.

Lower UST concentrations and higher CL were associated with a higher probability of active endoscopic disease (EHI > 50) (Figure 3A; *p* = 0.001; Figure 3B; *p* < 0.001, respectively) with greater changes in the probability of endoscopic active disease across the range of CL, as compared to concentrations.

## 4. Discussion

The clinical role of the use of TDM in the management of patients with IBD receiving UST therapy is still debated. Even though studies have found a correlation between lower UST drug concentrations and poor outcomes, this association has not been as robust as what has previously been seen with anti-TNF-α [6]. However, a better understanding of the PK properties of biologic drugs in IBD patients has provided the opportunity to explore other parameters that could potentially help understand mechanisms of non-response and even help optimize therapies. UST CL corresponds to the volume of serum cleared of the drug as a function of time and is, in part, an intrinsic patient characteristic, reflecting, for example, protein metabolism (e.g., its recirculation through the FcRn receptor) [33,34]). This is also in part related to the disease characteristics and the inflammatory burden where higher inflammation accelerates drug CL (protein-losing enteropathy) and consumption (target-mediated drug disposition).

Since our current study only included trough UST concentrations, we needed to define a set of Bayesian priors from a historical data set that was more extensively sampled to allow us to adequately estimate the UST PK in the current study. Due to the known differences between the solid-phase and HMSAs, we first re-parameterized the historical study by re-assaying its samples with the HMSA. We confirmed that UST concentrations measured using HMSA were higher than those measured using ELISA; because the impact of ATU was not evaluated in the European cohort, immunogenicity was estimated from a large database of >10,000 patient specimens from the CLIA PK laboratory. The analytical performances of the population PK model in estimating CL were below 10% for both repeatability and reproducibility, thus supporting its validity for testing in clinical PK practice.

Just as previously described with anti-TNF-α drugs, we found an association between prior biologic exposure and worse clinical outcomes [21]. In this study, the majority (>90%) of patients with previous biologic exposure never achieved clinical and biochemical remission, which is likely related to the fact this patient population had a more refractory disease phenotype and potentially individual characteristics that can negatively affect the PK of monoclonal antibodies. Whether the efficacy of drugs with a different molecular structure and metabolism (such as targeted small molecules) have a higher chance to work in this patient population is still unknown and warrants further investigation.

Through maintenance therapy, we also found that UST concentrations were poorly associated with disease activity, which is consistent with the results of previous meta-analyses [6]. This reflects the limitations of using only drug concentration to perform TDM in these patients. In fact, the dose response relationship revealed that, across the dynamic range of the concentrations, there was little change in the probability of achieving remission (Figure 2). In contrast, we found that UST CL was better correlated with outcomes when compared to trough UST concentrations. The longitudinal analyses of our data revealed that CL was the only PK parameter associated with disease control, with large changes in the probability of remission across the range of CL observed in the study. This is comparable to what we have already seen with anti-TNF-α [20,21], implicating some degree of generalizability across monoclonal antibodies.

We also found that the non-invasive seroproteomic panel of endoscopic active disease (EHI) was better associated with higher CL, as compared to concentration (Figure 3), a notion that is consistent with the hypothesis that CL reflects both patients and disease characteristics, as noted above.

Our study has notable strengths, including the prospective design and the longitudinal collection of specimens throughout the maintenance period. However, we do acknowledge a limitation. Among our outcome measures, endoscopic disease activity was not available; to address this, we did measure the EHI as a validated surrogate marker of endoscopically active disease in CD.

Given the limitations of UST concentrations alone in guiding clinical choice with respect to dose and interval optimization, CL represents a novel metric, which can serve as an important clinically available tool to aid in managing patients on UST and other monoclonal antibodies [20]. CL could be incorporated into routine TDM and dose optimization decisions for UST with an opportunity in clinical practice to attempt to recapture response with dose intensification in the presence of lower concentrations (i.e., below 4.5 µg/mL) [15] as seen with HMSA. Prospective and interventional studies will be essential to further study the potential clinical utility.

## 5. Conclusions

In conclusion, our report demonstrates that UST CL is better associated with IBD outcomes as compared to UST concentrations alone, illustrating a potential role for the assessment of CL in the management of patients with IBD, especially in the setting of non-response. Cl could be incorporated in clinical practice where higher CL would predict a higher likelihood of poor disease control leading to early dose escalation. Interventional trials addressing the clinical utility of CL in optimizing UST are warranted. Randomized trials looking into the utility of measuring UST CL and how this tool can help improve short- and long-term outcomes could ultimately establish UST TDM as a standard of care.

## Figures and Tables

**Table 1:** Population PK model reparameterization and Bayesian priors.

Parameter	Training Cohort ELISA (n = 308)	Training Cohort HMSA (n = 308)	Prometheus PK Database HMSA (n = 11,372)	Final Bayesian Priors
Ka (day^−1^)	0.142 (fixed)	0.142 (fixed)	0.142 (fixed)	0.142 (fixed)
Bioavailability (F)	0.624	0.735	0.735 (fixed)	0.735 (fixed)
CL_pop (L/day)	0.235	0.161	0.161 (fixed)	0.161 (fixed)
V1_pop (L)	5.05	3.72	3.72 (fixed)	3.72 (fixed)
Q (L/day)	0.0436	0.0322	0.0322 (fixed)	0.0322 (fixed)
V2 (L)	0.748	0.393	0.393 (fixed)	0.393 (fixed)
IIV on F	37.3%	20.3%	NA	NA
IIV of CL	31.2%	30.4%	30.4% (fixed)	30.4% (fixed)
effect of ATU on CL *	NA	NA	0.566 (8.65%)	0.566 (fixed)
effect of ALB on CL *	−1.21	−0.887	NA	−0.887 (fixed)
Additive error (µg/mL)	0.272	0.495	0.495 (fixed)	0.495 (fixed)
Proportional error (%)	13.6%	11.0%	11.0% (fixed)	11.0% (fixed)

* Covariate models: 1: CL = CL_population_ × exp (β × ATU = Positive); 2: CL = CL_population_ × (Albumin/4.0)^β^; IIV: Inter-individual variability. NA: not applicable.

**Table 2:** Repeatability (intra-day).

	UST (µg/mL)		ALB (g/dL)		CL (L/Day)	
Specimen	Mean ± SD	CV%	Mean ± SD	CV%	Mean ± SD	CV%
#1	7.3 ± 0.3	4%	3.9 ± 0.1	2%	0.203 ± 0.004	2%
#2	<0.9	NA	2.6 ± 0.1	3%	0.310 ± 0.003	1%
#3	6.1 ± 0.3	5%	3.5 ± 0.2	5%	0.122 ± 0.003	3%
#4	11.9 ± 0.5	4%	4.3 ± 0.1	2%	0.073 ± 0.002	3%
#5	2.0 ± 0.1	4%	2.6 ± 0.2	6%	0.226 ± 0.005	2%
#6	>25.0	NA	3.6 ± 0.0	1%	0.066 ± 0.000	1%
#7	16.4 ± 0.6	4%	4.2 ± 0.1	1%	0.078 ± 0.002	3%
#8	15.8 ± 0.7	5%	3.6 ± 0.1	2%	0.123 ± 0.002	3%

NA: not applicable, below LLOQ or above ULOQ.

**Table 3:** Reproducibility (inter-day).

	UST (µg/mL)		ALB (g/dL)		CL (L/Day)	
Specimen	Mean ± SD	CV%	Mean ± SD	CV%	Mean ± SD	CV%
#1	7.7 ± 0.4	5%	4.0 ± 0.1	1%	0.197 ± 0.005	3%
#2	<0.9	NA	2.7 ± 0.1	3%	0.307 ± 0.004	1%
#3	6.7 ± 0.2	4%	3.5 ± 0.1	4%	0.115 ± 0.002	2%
#4	12.5 ± 0.3	2%	4.4 ± 0.1	2%	0.070 ± 0.001	1%
#5	2.1 ± 0.2	8%	2.5 ± 0.1	2%	0.225 ± 0.008	4%
#6	>25.0	NA	3.6 ± 0.1	3%	0.066 ± 0.001	2%
#7	17.8 ± 0.7	4%	4.3 ± 0.1	3%	0.073 ± 0.002	3%
#8	17.7 ± 0.2	1%	3.7 ± 0.2	4%	0.113 ± 0.001	1%

NA: not applicable, below LLOQ or above ULOQ.

**Table 4:** Patients characteristics.

Parameter	Estimate
Number of subjects, n	83
Female gender, n (%)	43 (52%)
Crohn’s disease diagnosis (vs. ulcerative colitis), n (%)	78 (94%)
Number of cycles per patient, median [IQR]	3 [1–3]
Age: ≤16 (A1)/17–40 (A2)/>40 (A3)	23/42/18
**Crohn’s disease**	
Location: Terminal Ileum (L1)/Colonic (L2)/Ileocolonic (L3)	12/15/51
Behavior: Non-strict, non-pen. (B1)/Stricturing (B2)/Penetrating (B3)	26/32/20
Perianal disease, n (%)	25 (32%)
**Ulcerative Colitis**	
Proctitis (E1)/Left sided colitis (E2)/Pancolitis (E3)	1/2/2
**Treatment**	
Previous exposure to biologics, n (%)	89.0% (74/83)
Concomitant Immunomodulators, n (%)	13 (16%)
Follow-up time from baseline (days), Median [IQR]	279 (210–351)
**UST PK and outcomes (all cycles)**	
Interdose interval (days), Median [IQR]	56 [42–56]
Interval between specimen collection (days)	56 [30–57]
UST concentrations at trough (µg/mL), Median [IQR]	5.0 [3.0–8.7]
UST concentrations >4.5 µg/mL at the trough, % (n/N)	54% (167/312)
ATU positive status, % (n/N)	0% (0/312)
Clearance (L/day), Median [IQR]	0.157 [0.120–0.190]
Clearance < 0.161 L/day, % (n/N)	58% (182/312)
Albumin (g/dL), Median [IQR]	3.8 [3.5–4.1]
Clinical and biochemical remission, % (n/N)	26% (84/312)
EHI score (n = 303 cycles), Median [IQR]	33 [23–43]
EHI score <20 (endoscopic remission), % (n/N)	20.1% (61/303)
EHI score >50 (endoscopic active disease), % (n/N)	17.8% (54/303)
**UST PK and outcomes (patient level)**	
Clinical and biochemical remission, never achieved, % (n/N)	55.4% (46/83)
Clinical and biochemical remission: sometimes achieved, % (n/N)	36.1% (30/83)
Clinical and biochemical remission: always achieved, % (n/N)	8.4% (7/83)

Results are reported as median with interquartile range (IQR) or percentage, as appropriate.

## Data Availability

No data sharing is available.

## Supplementary Materials

The following supporting information can be downloaded at https://www.mdpi.com/article/10.3390/pharmaceutics17020187/s1. Table S1: Estimates from logistic regression models.
