# Supplementary material for: Ustekinumab Drug Clearance Is Better Associated with Disease Control than Serum Trough Concentrations in a Prospective Cohort of Inflammatory Bowel Disease
Source: Pharmaceutics. 2025 Feb 2;17(2):187. doi: 10.3390/pharmaceutics17020187 (PMC11859385; doi:10.3390/pharmaceutics17020187)
Supplement: Supplementary file 1 [file pharmaceutics-17-00187-s001.zip › pharmaceutics-3385963-supplementary.pdf]

SUPPLEMENTARY MATERIALS

USTEKINUMAB DRUG CLEARANCE IS BETTER ASSOCIATED WITH DISEASE CONTROL THAN SERUM TROUGH CONCENTRATIONS IN PROSPECTIVE COHORT OF INFLAMMATORY BOWEL DISEASE

Table S1: Estimates from logistic regression models

All estimates were calculated using Monolix

Model	Estimates
Clinical and biochemical remission with prior biologics as covariate and time as regressor	θ_{pop}=+0.006±0.85<br>$\theta_{prior\ biologics}$= -2.48±0.87 (p=0.004)<br>$\theta_{time}$ =+0.0024±0.0017 (p=0.158)
Clinical & biochemical remission with prior biologics as covariate and UST concentration as regressor	θ_{pop}=0.22±0.84<br>$\theta_{prior\ biologics}$= -2.36±0.85 (p=0.005)<br>$\theta_{conc}$ =+0.024±0.045 (p=0.533)
Clinical & biochemical remission with prior biologics as covariate and UST CL as regressor	θ_{pop}=+2.29±0.88<br>$\theta_{prior\ biologics}$= -2.02±0.81 (p=0.013)<br>$\theta_{CL}$ =-13.5±3.7 (p<0.001)
EHI>50 with prior biologics as covariate and UST concentration as regressor	θ_{pop}=-1.85±1.55<br>$\theta_{prior\ biologics}$= -0.52±1.50 (p=0.731)<br>$\theta_{conc}$ =-0.072±0.045 (p=0.001)
EHI>50 with prior biologics as covariate and UST CL as regressor	θ_{pop}=-6.29±1.45<br>$\theta_{prior\ biologics}$= +0.639±1.45 (p=0.659)<br>$\theta_{CL}$ =18.0±3.2 (p<0.001)

$logit(P)= \theta_{pop} + cov_1*\theta_{cov1} + cov_2*\theta_{cov2}.$
